# A preliminary study on the monitoring of mixed venous oxygen saturation through the left main bronchus

**DOI:** 10.1186/cc3914

**Published:** 2005-12-06

**Authors:** Xiang-rui Wang, Yong-jun Zheng, Jie Tian, Zheng-hong Wang, Zhi-ying Pan

**Affiliations:** 1 Professor of anesthesiology, Department of Anesthesiology, Renji Hospital affiliated to Shanghai Second Medical University, 1630 Dongfang Road, Shanghai, 200127, China; 2 Resident, Department of Anesthesiology, Renji Hospital affiliated to Shanghai Second Medical University, 1630 Dongfang Road, Shanghai, 200127, China

## Abstract

**Introduction:**

The study sought to assess the feasibility and accuracy of measuring mixed venous oxygen saturation (SvO_2_) through the left main bronchus (SpO_2trachea_)

**Methods:**

Twenty hybrid pigs of each sex were studied. After anesthesia, a Robertshaw double-lumen tracheal tube with a single-use pediatric pulse oximeter attached to the left lateral surface was introduced toward the left main bronchus of the pig by means of a fibrobronchoscope. Measurements of SpO_2trachea _and oxygen saturation from pulmonary artery samples (SvO_2blood_) were performed with an intracuff pressure of 0 to 60 cmH_2_O. After equilibration, hemorrhagic shock was induced in these pigs by bleeding to a mean arterial blood pressure of 40 mmHg. With the intracuff pressure maintained at 60 cmH_2_O, SpO_2trachea _and SvO_2blood _were obtained respectively during the pre-shock period, immediately after the onset of shock, 15 and 30 minutes after shock, and 15, 30, and 60 minutes after resuscitation.

**Results:**

SpO_2trachea _was the same as SvO_2blood _at an intracuff pressure of 10, 20, 40, and 60 cmH_2_O, but was reduced when the intracuff pressure was zero (*p *< 0.001 compared with SvO_2blood_) in hemodynamically stable states. Changes of SpO_2trachea _and SvO_2blood _corresponded with varieties of cardiac output during the hemorrhagic shock period. There was a significant correlation between the two methods at different time points.

**Conclusion:**

Measurement of the left main bronchus SpO_2 _is feasible and provides similar readings to SvO_2blood _in hemodynamically stable or in low saturation states. Tracheal oximetry readings are not primarily derived from the tracheal mucosa. The technique merits further evaluation.

## Introduction

The saturation of haemoglobin with oxygen in the pulmonary artery is known as the mixed venous oxygen saturation (SvO_2_), which reflects the balance between the amount of oxygen delivered to the tissues and how much is used. It enables an estimate of the oxygen supply/demand balance to be made and hence enhances our comprehension of physiological concepts of hemodynamics and tissue oxygenation in critically ill patients. However, the routine measurement of SvO_2 _requires the placement of a pulmonary artery catheter (PAC), which may not always be feasible. Furthermore, a substantial review of literature suggests at present that the use of PAC may lead to an overall increase in morbidity and mortality in critically ill patients [1,2], stimulating the quest for a micro-invasive tool for assessing SvO_2_.

Pulse oximetry has been widely adopted in anesthesia and critical care medicine to provide noninvasive information about arterial oxygen saturation (SaO_2_). Several studies have demonstrated that oximeters placed in deep, vessel-rich areas such as the esophagus [3], pharynx [4], and trachea [5] seemed to provide more accurate readings than superficial oximetry. The tissue being sampled was once assumed to be the surrounding mucosa [3], but recent studies have shown that the signals were derived primarily from deeper tissues, such as underlying large vessels around the esophagus and trachea [5,6].

**Figure 1:**
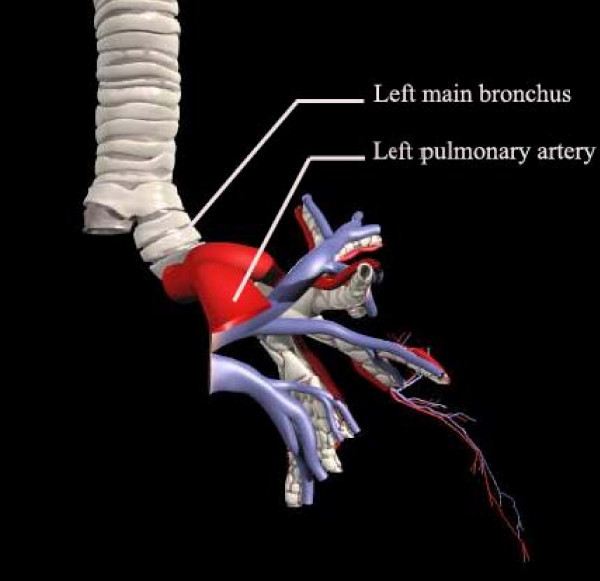
Anatomic relationship between the left main bronchus and the left pulmonary artery.

The pulmonary artery lies close to the bronchus, with nothing but some connective tissues between them, raising the possibility that an appropriately located and directed bronchial oximetry probe might be able to derive oximetry readings from mixed venous blood (Figure 1). The present study was undertaken to test the feasibility of measuring SvO_2 _through the left main bronchus (SpO_2trachea_), and to compare SpO_2trachea _with oxygen saturation from pulmonary artery samples (SvO_2blood_) in a healthy hybrid pig to improve our understanding of the hypothesis that bronchial oximetry readings are derived primarily from the pulmonary artery, not from the tracheal mucosa. Furthermore, the stability and accuracy of SpO_2trachea _were evaluated by assessing the impact of altered cardiac output on tracheal SpO_2 _in hemorrhagic shock status.

## Materials and methods

### Anesthesia and surgical preparation

The study was approved by the rules of Veterinary Medicine and Animal Care. After 12 hours of fasting, 20 Shanghai hybrid pigs (Shanghai University, Shanghai, China) of both sexes, weighing 50.7 ± 3.2 kg, were premedicated intramuscularly with ketamine (20 mg kg^-1^) and atropine (0.04 mg kg^-1^). Anesthesia was maintained by the intermittent application of pentothal sodium (2.5%) and diazepam. After endotracheal intubation of a Robertshaw double-lumen tracheal tube (details are given in the section on fabrication of the measuring catheter and intubation), the animals were ventilated mechanically with oxygen. The ventilation rate was 16 breaths min^-1^, and the respiratory tidal volume was set to 10 to 15 ml kg^-1 ^body weight to adjust the end-expiratory partial pressure of CO_2 _to 4.5 to 6.0 kPa. The Inspire:Expire (I:E) ratio was 1:2. Respiratory rates, tidal volume and concentrations of oxygen and carbon dioxide were adjusted in accordance with periodic blood gas analysis to keep adequate blood pH. The right femoral artery was cannulated with a 22-gauge catheter connected to a pressure sensor to measure the mean artery pressure. The left femoral vein was cannulated with a 7F Swan–Ganz catheter, which was positioned according to the wave form, for intermittent sampling of pulmonary arterial blood for blood gas analysis. The right internal jugular vein was cannulated with a catheter to provide a venous line for infusion and anesthesia. Throughout the experiments, all animals received a Ringer lactate solution infusion at a rate of 10 ml kg^-1 ^h^-1^. Electrocardiograph, heart rate and mean artery pressure were monitored continuously.

### Refitting the oximetry probe, and stability test

Because a pulse oximeter stops working when in contact with water or another fluid, it should be waterproofed before use. The processing of disposable single-use pediatric pulse oximeters (Datex Medical Instrumentation, Helsinki, Finland) adopted in our experiments was as follows. First the fixed membrane was removed, the light emitter and sensor were exposed, then a surface coat of medical silica gel (provided by Shanghai Latex Institute) was applied, leaving it to solidify at normal temperature for 72 hours. Medical silica gel is made from pure silica gel with very thin texture. It is capable of forming a fine surface coating and can withstand a certain level of friction and tension after full solidification at normal temperature. Pulse oximetry of the tongue was obtained with both the refitted oximetry probe and the original probe. The readings were compared to test the stability and accuracy of the refitted probe.

### Fabrication of the measuring catheter, and intubation

**Figure 2:**
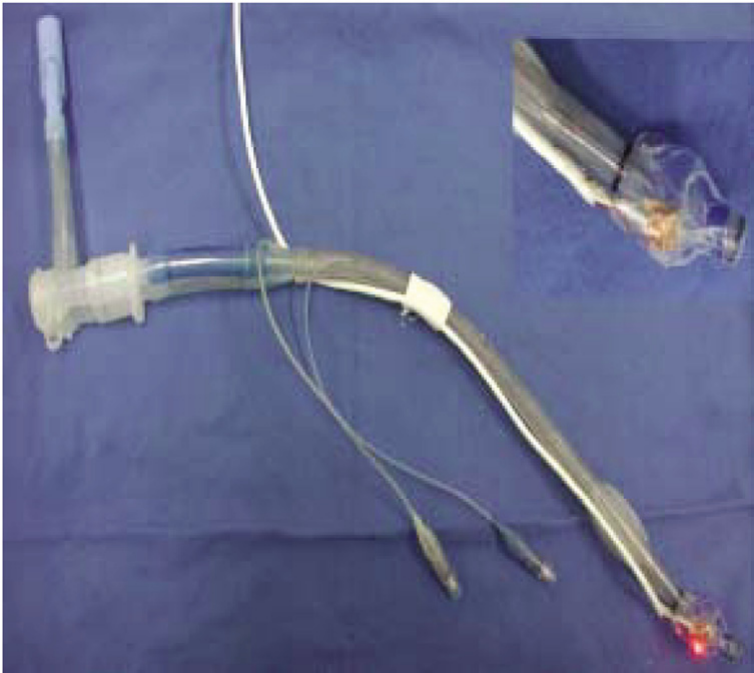
The Robertshaw double-lumen tracheal tube attached to a single-use pediatric pulse oximeter.

After inflation of the left lateral cuff portion of a Robertshaw double-lumen tracheal tube (37F), the light emitter and sensor of the waterproof oximeter were fixed along the longitudinal axis of the tracheal tube, and the infrared probe of the light emitter and the light-sensitive surface of the light sensor were faced in the same direction. The sensor was wrapped with copper foil except for a small window to expose the light-sensitive plate. A distance of 1 cm was left between the two terminals. Then the oximeter probe was fixed to the tube with a medical membrane, with two holes in the position of the light emitter and sensor to avoid any possible interference, as shown in Figure 2.

**Figure 3:**
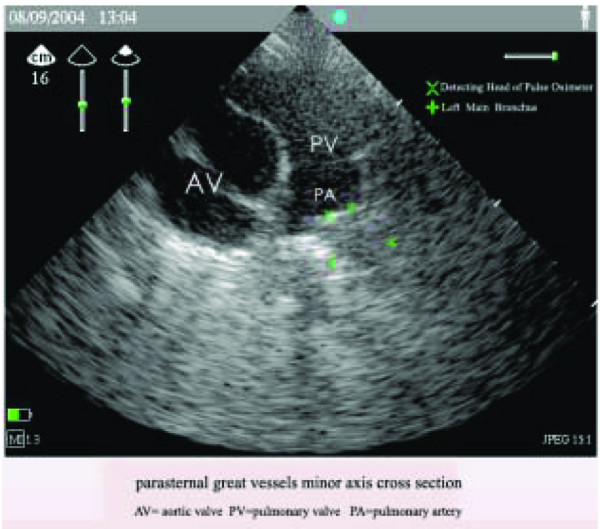
The position of the oximeter confirmed by ultrasound. A minor-axis cross-section of parasternal great vessels is shown, and is representative of 20 subjects. AV, aortic valve; PA, pulmonary artery; PV, pulmonary vein.

After anesthesia, the head and neck of the pig were positioned in the midline, with the occiput on a pillow 7 cm in height. The tracheal tube was inserted into the left main bronchus under the guidance of a pediatric fibrobronchoscope, and positioned at adequate depth and in an appropriate direction (the pilot open chest study had proved that a depth of 2 to 3 cm was adequate and that an appropriate direction was 15 to 20° left-leaning to the midline) to ensure that it was on the opposite side of the left pulmonary artery. Then the oximeter was connected to a monitor (Datex AS/3; Datex Medical Instrumentation) that had been previously checked and calibrated to ensure that it gave the same reading when attached to the same probe. The tracheal tube was fixed once the oxygen saturation curve had become a sine wave, and the position of the oximeter was confirmed by ultrasound and chest radiology (Figure 3).

### Changes in SvO_2 _with intracuff pressure

SpO_2trachea _was measured during a hemodynamically stable period of anesthesia. Readings were allowed to stabilize for two minutes before they were recorded. At the same time pulmonary arterial blood was collected and analyzed to measure SvO_2blood _(Serie 800; Chiron Diagnostics GmbH, Salzburg, Austria). The arterial blood gas monitor was accurate to 0.01% (SaO_2_) and calibrated before each case. Readings were taken with an intracuff pressure of 0, 10, 20, 40, and 60 cmH_2_O. The intracuff pressure was set with a digital cuff pressure monitor (Digital P-V Gauge™; Mallinckrodt Medical). One set of observations was obtained in each animal at each cuff pressure. All observations were made in a hemodynamically stable period.

### Changes in SvO_2 _in hemorrhagic shock status

The same 20 pigs were used in the present study. After instrumentation, pigs were allowed to equilibrate for 30 minutes; they then underwent a standardized controlled hemorrhage to a mean artery pressure of 40 mmHg and were maintained at this level for 60 minutes. During hemorrhage, the blood was stored in a closed reservoir primed with sodium citrate and pig heparin to inhibit clot formation. At the end of 60 minutes, animals were resuscitated with the preserved shed blood, which was withdrawn from the pig to induce hypotension, and an equal volume of lactated Ringers to restore the baseline mean artery pressure. Cardiac output was assessed by the thermal dilution method during the procedure. The intracuff pressure was kept at 60 cmH_2_O. SpO_2trachea _and SvO_2blood _were measured at the pre-shock period, immediately after the onset of shock, 15 and 30 minutes after shock, and 15, 30 and 60 minutes after resuscitation.

### Statistical analysis

Results are reported as means ± SEM and analyzed with a pair-matching *t *test and linear regression. To compare the accuracy of the new method, Bland–Altman plots were used. *p *< 0.05 was considered statistically significant.

## Results

### Stability and accuracy of the refitted oximetry probe

Pulse oximetry of the tongue was obtained with both the refitted oximetry probe (SpO_2refit_) and the original probe (SpO_2origin_) to test the stability and accuracy of the refitted probe. SpO_2refit _was similar to SpO_2origin _when the probe contacted tightly with the tongue (*p *> 0.05). The readings did not vary with changing intracuff pressure, and there was significant correlation between the two kinds of probe (*p *< 0.01; Table 1). However, SpO_2refit _was significantly lower than SpO_2origin _if there were spaces between the probe and the tongue (*p *< 0.001).

### Correlations between SpO_2trachea _and the intracuff pressure in normal situation

**Figure 4:**
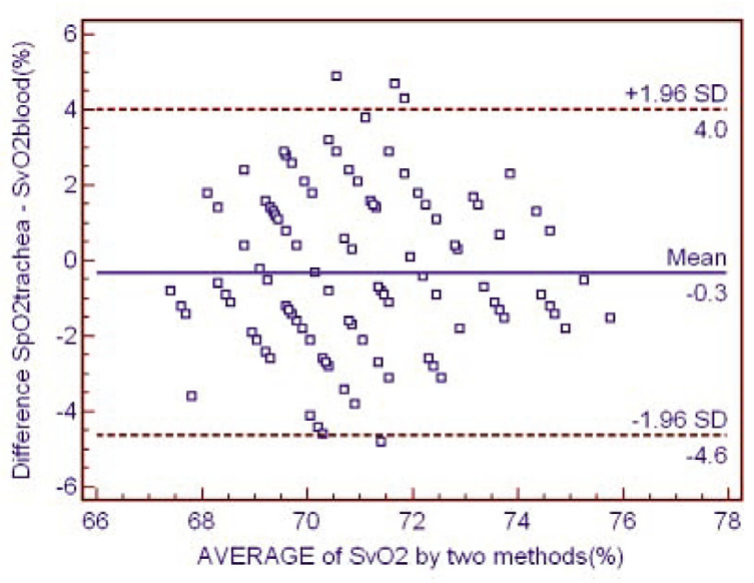
The accuracy of the new method in hemodynamically stable status. Shown is a Bland–Altman graph comparing the difference between mixed venous oxygen saturation through the left main bronchus (SpO_2trachea_) and oxygen saturation from pulmonary artery samples (SvO_2blood_) versus the mean oxygen saturation by the 'gold standard' and the new method in hemodynamically stable status.

The age and weight ranges of the pigs were 6–8 months and 45–55 kg, respectively. The male:female ratio was 8:12. The mean (range) core temperature during the readings was 36.4°C (36.0 to 36.9°C) with the room temperature maintained at 21°C. SpO_2trachea _was the same as SvO_2blood _at an intracuff pressure of 10 to 60 cmH_2_O with no significant differences (*p *> 0.05) but significant correlations (*p *< 0.01) between each other (Tables 2 and 3). Values of SvO_2blood _did not vary with changing intracuff pressure, but SpO_2trachea _was lower when intracuff pressure was zero. There were significant differences between them (*p *< 0.001; Tables 2 and 3). Bland–Altman graphs for SpO_2trachea _versus SvO_2blood _are presented in Figure 4.

### Changes in SpO_2trachea _in hemorrhagic shock status and correlations between SpO_2trachea _and SvO_2blood_

**Figure 5:**
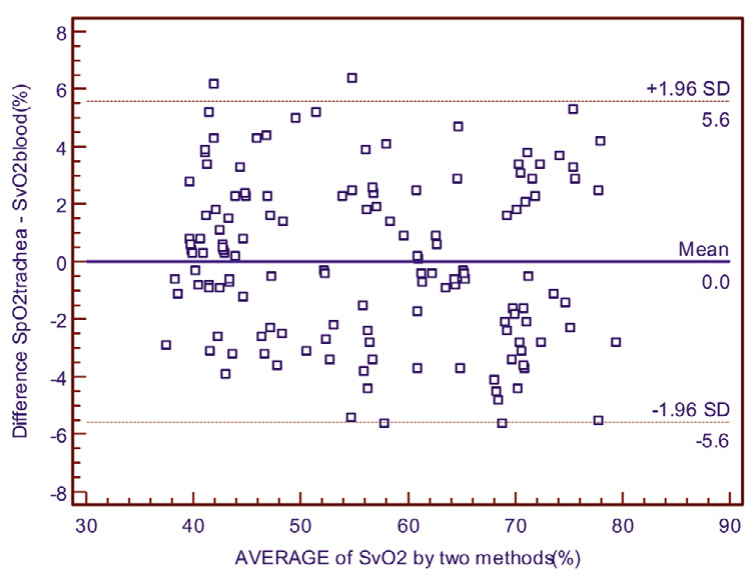
The accuracy of the new method in hemorrhagic shock status. Shown is a Bland–Altman graph comparing the difference between mixed venous oxygen saturation through left main bronchus (SpO_2trachea_) and oxygen saturation from pulmonary artery samples (SvO_2blood_) versus the mean oxygen saturation by the 'gold standard' and the new method in hemorrhagic shock status.

With the intracuff pressure maintained at 60 cmH_2_O, changes in SpO_2trachea _and SvO_2blood _were due to variations in cardiac output during the hemorrhagic shock period (Table 4). There was significant correlation between SpO_2trachea _and SvO_2blood _(*p *< 0.01; Table 5). Bland–Altman analysis revealed excellent accordance between the two methods, with only few points located outside the 'limits of agreement' area (Figure 5).

## Discussion

SvO_2 _reflects the balance between oxygen delivery and demand. It decreases when oxygen delivery has been compromised or systemic oxygen demands have exceeded supply. Its ability to give a real-time indication of tissue oxygenation makes it a preferred parameter for monitoring the adequacy of hemodynamics. In comparison with traditional parameters such as arterial oxygen saturation and cardiac output, SvO_2 _allows a more precise understanding of the adequacy of cardiac and pulmonary function. Declines in SvO_2 _precede the onset of inadequate myocardial function, shock, or the development of arrhythmias, even though vital signs may be normal. Its use as an end point for determining the adequacy of hemodynamics (blood pressure, cardiac output/cardiac index), measurement of right to left shunt, and prediction of potential hemodynamic instability makes this parameter invaluable for the knowledgeable clinician. There is now evidence that the timing of diagnostic and therapeutic intervention using this technology may be a critical determinant of outcome [7].

The PAC, otherwise known as the Swan–Ganz catheter, was developed by cardiologists HJC Swan and William Ganz in 1970. It is a flexible balloon-tipped flow-directed catheter that, when inserted via central venous access, can be guided into a branch of the pulmonary artery. Its ability to provide continuous measurements of SvO_2 _in critically ill patients makes its use invaluable in the provision of quality medical care. However, controversy surrounding the efficacy and safety of the PAC has been going on for many years. The complications can be categorized as those of the initial venous cannulation (subclavian or carotid artery laceration, pneumothorax, thoracic duct laceration, phrenic nerve injury, and air embolism) and those due to the catheter itself (*ie*, arrhythmias, infection, valvular damage, thrombosis, pulmonary infarction, and rupture of the pulmonary artery). At the same time, the device requires a trained operator and is time-consuming. Moreover, it is expensive, bringing high healthcare costs.

There is therefore a powerful need for a method to measure SvO_2 _more safely. Other researchers have developed the technique of deriving oximetry readings of arterial blood through the trachea, or right and left ventricular oximetry through the esophagus [5,8]. The pulmonary artery is known to lie just proximal to the left bronchus. This evaluation of the anatomy made it practical to measure oximetry readings from the mixed venous circulation through the left main bronchus. However, so far no such studies have been reported. The present study establishes the first investigation to assess SvO_2 _microinvasively according to the above anatomic and technological bases.

Waterproofing is crucial for the proper function of oximeters in the humid environment of the trachea. Our experiment employed medical silica gel as a surface coat, because silica gel is waterproof and is nontoxic to humans. It can solidify fully at normal temperature, thus avoiding potential damage to the oximeter caused by thermal treatment. Moreover, it can endure a certain level of friction and tension after solidification. Because the pulmonary artery and the bronchus run nearly parallel, with sufficient overlapping area in the longitudinal direction, the light emitter and sensor of the oximeter are affixed along the same direction on the tracheal tube. As a result, the probe turned from a penetrating model (the light emitter and sensor being aligned opposite each other) into a reflecting model (the two terminals lying side by side). Experimental results indicate that the optimum distance between the emitter and sensor should be close to 1 cm. If the two terminals are too close, transmitting signals will be attenuated, which will affect the stability and accuracy of the data. Conversely, an increase in distance will negatively affect the reception efficiency of the infrared reflection signal.

Despite the above changes to the oximetry probe, high-quality signals were still available. We found that SpO_2refit _of the tongue was accurate at different inspiratory oxygen concentrations, in different head and neck positions, and over a prolonged period, suggesting good stability and sensitivity of the refitted probe.

The ability to localize the oximetry probe accurately is pivotal to the experiment. An experiential position 2 to 3 cm deep in the bronchus and an orientation of 15 to 20° left-leaning to the midline for the tracheal tube was found in our pilot study. To ensure that the tube was advanced to the optimal location, the animal should be fixed beforehand, and the position of the tube should be confirmed by electrocardiography.

Supported by the foregoing statement, our data showed that the reading of SpO_2trachea _was close to SvO_2blood _in stable physiological situations at 10 to 60 cmH_2_O cuff pressure. The readings obtained at zero cuff pressure were probably low because of a lack of contact between the probe and the trachea. The SpO_2trachea _was thought not to be derived primarily from the tracheal mucosa, because tracheal mucosal perfusion ceases when the intracuff pressure exceeds 50 cmH_2_O, and there was no decrease in the accuracy of SpO_2trachea _with increasing intracuff pressure. The blood flowing through the left pulmonary artery was speculated to be the mass of tissue sampled by the tracheal oximetry probe. At the same time, our study showed that SpO_2trachea _was consistent with SvO_2blood _in low cardiac output status during the hemorrhagic shock period. This measurement demonstrated that the precision of measuring SvO_2 _through the left main bronchus was not influenced in a pathological state, suggesting great reliability of this technique in operation and for patients in intensive care units. Although ventilation with a double-lumen tube is itself an invasive procedure, its advantage in causing much fewer lesions than PAC cannulation, and in avoiding the multiple complications that accompany the PAC device, makes this technique particularly appropriate for critically ill patients.

However, several limitations of the present investigation should be noted. First, our device was homemade, with the oximeter probe fixed to the endoscope by tape. Damage to the mucosa of the trachea is possible, and accidental inhalation would occur if the probe exfoliated. Furthermore, to reduce complications, a small tracheal tube and thin wire were required. However, it would be possible to incorporate the oximeter within the cuff and the wire within the tube and in so doing to reduce the complication of damage or accidental inhalation and allow a larger tube to be used to decrease the risk of trauma. Secondly, there were difficulties with locating the probe in the left bronchus. In addition to adjusting the tube repeatedly, ultrasound is required to confirm the position of the oximeter. The technique for location merits further investigation.

## Conclusion

Measurement of SpO_2 _via the left main bronchus is feasible and provides similar readings to SvO_2blood _in both hemodynamically stable status and hemorrhagic shock status. Tracheal oximetry readings are not derived primarily from the tracheal mucosa. This technique is capable of providing continuous and microinvasive measurements of SvO_2 _despite the difficulty in achieving proper location of the probe. Further improvement is required for convenience of operation.

## Key messages

• 	An appropriately located and directed bronchial oximetry probe is able to derive oximetry readings from the pulmonary artery, because the artery lies in close proximity to the bronchus with only some connective tissues in between, thus providing a microinvasive tool for the assessment of mixed venous oxygen saturation (SvO_2_).

• 	The mixed venous oxygen saturation via the left main bronchus (SpO_2trachea_) was thought not to derive primarily from the tracheal mucosa, because it was lower than the oxygen saturation from pulmonary artery samples (SvO_2blood_) at zero cuff pressure.

• 	SpO_2trachea _was the same as SvO_2blood _in hemodynamically stable status.

• 	Sp_O2trachea _also provides similar readings to SvO_2blood _in hemorrhagic shock status, suggesting great reliability of this technique in operation and for patients in intensive care units.

## Abbreviations

PAC = pulmonary artery catheter; SaO_2 _= arterial oxygen saturation; SpO_2origin _= pulse oximetry obtained with the original oximetry probe; SpO_2refit _= pulse oximetry obtained with the refitted oximetry probe; SpO_2trachea _= SvO_2 _through the left main bronchus; SvO_2 _= mixed venous oxygen saturation; SvO_2blood _= oxygen saturation from pulmonary artery samples.

## Competing interests

The study was funded by 'The Third Period of Hundred People Project, Shanghai City'.

## Authors' contributions

XW conceived the study, participated in the design and execution of the study, and finalized and revised the manuscript. YZ participated in the animal experiments, performed the statistical analysis, and was involved in drafting the manuscript. JT participated in study design, interpretation of the results, and writing the manuscript. ZW and ZP participated in the animal experiments. All authors read and approved the final manuscript.

## Figures and Tables

**Table 1:** Comparisons of pulse oximetry measurements on the tongue with the original and refitted oximetry probes

Concentration of inspiratory oxygen (%)	*n*	Oxygen saturation (%)		Correlation coefficient (*r*)
			
		SpO_2refit_	SpO_2origin_	
100	10	100	100	1.0
21	10	93.2 ± 2.4 (92–96)	93.4 ± 2.7 (91–96)	0.95
10	10	81.5 ± 2.2 (77–84)	81.1 ± 2.5 (78–85)	0.94

Values are means ± SEM (range). SpO_2origin_, pulse oximetry obtained with the original oximetry probe; SpO_2refit_, pulse oximetry obtained with the refitted oximetry probe.

**Table 2:** Oxygen saturation measurements in physiological states

Intracuff pressure (cmH_2_O)	*n*	Oxygen saturation (%)	
		
		SpO_2trachea_	SvO_2blood_
0	20	70.2 ± 6.2 (57–76)	74.4 ± 4.3 (62.6–76.4)
10	20	74.2 ± 4.7 (62–77)	74.4 ± 4.4 (62.5–76.9)
20	20	74.2 ± 4.8 (62–77)	74.3 ± 4.3 (62.4–76.7)
40	20	74.2 ± 4.6 (61–76)	74.4 ± 4.3 (62.3–76.9)
60	20	74.2 ± 4.6 (62–77)	74.3 ± 4.4 (62.5–77.1)
Overall	100	72.5 ± 6.8 (57–77)	74.4 ± 6.3 (61.9–77.2)
Overall excluding 0 cmH_2_O	80	74.2 ± 4.2 (61–77)	74.4 ± 4.3 (61.2–77.6)

Values are means ± SEM (range). SpO_2trachea_, mixed venous oxygen saturation measured through the left main bronchus; SvO_2blood_, oxygen saturation from pulmonary artery samples.

**Table 3:** Between-method statistical comparisons for the oxygen saturation measurement (SpO_2trachea _versus SvO_2blood_)

Intracuff pressure (cmH_2_O)	*n*	MD (%)	SD	SEM	LOA	SEL
0	20	4.87	3.10	0.73	-1.33 to 11.07	1.201
10	20	0.25	0.97	0.21	-1.69 to 2.19	0.376
20	20	0.22	0.89	0.19	-1.56 to 2.00	0.345
40	20	0.31	0.66	0.14	-1.01 to 1.63	0.256
60	20	0.17	0.74	0.18	-1.31 to 1.65	0.287
Overall	100	1.26	2.39	0.25	-3.52 to 6.04	0.414
Overall excluding 0 cmH_2_O	80	0.24	0.68	0.17	-1.12 to 1.6	0.132

LOA, limits of agreement (MD ± 1.96SD); MD, mean difference; SD, standard deviation of the difference; SEL, standard error of limit; SEM, standard error of the mean difference; SpO_2trachea_, mixed venous oxygen saturation measured through the left main bronchus; SvO_2blood_, oxygen saturation from pulmonary artery samples.

**Table 4:** Changes in SpO_2trachea _and SvO_2blood _in hemorrhagic shock status

Time	*n*	Oxygen saturation (%)	
		
		SpO_2trachea_	SvO_2blood_
Pre-shock period	20	74.6 ± 4.5 (62–78)	74.3 ± 4.7 (62.6–76.8)
Immediately after onset of shock	20	74.2 ± 4.3 (60–78)	74.8 ± 4.6 (61.9–77.2)
15 min after shock	20	61.2 ± 4.8 (52–67)	61.7 ± 4.3 (52.4–68.2)
30 min after shock	20	42.2 ± 4.6 (41–54)	42.8 ± 4.7 (41.3–55.9)
15 min after resuscitation	20	51.8 ± 4.6 (49–63)	51.3 ± 4.4 (49.5–62.6)
30 min after resuscitation	20	64.5 ± 6.8 (57–77)	64.2 ± 6.3 (57.9–77.2)
60 min after resuscitation	20	74.2 ± 4.2 (61–77)	74.4 ± 4.3 (61.2–77.6)

Values are means ± SEM (range). SpO_2trachea_, mixed venous oxygen saturation measured through the left main bronchus; SvO_2blood_, oxygen saturation from pulmonary artery samples.

**Table 5:** Between-method statistical comparisons for oxygen saturation measurements in hemorrhagic shock status (SpO_2trachea _versus SvO_2blood_)

Time	*n*	MD (%)	SD	SEM	LOA	SEL
Pre-shock period	20	-0.845	3.065	0.685	-6.975 to 5.285	1.187
Immediately after onset of shock	20	0.495	3.014	0.674	-5.533 to 6.523	1.167
15 min after shock	20	-0.165	3.210	0.718	-6.585 to 6.255	1.243
30 min after shock	20	-1.275	2.759	0.617	-6.793 to 4.243	1.069
15 min after resuscitation	20	-0.315	1.509	0.3374	-3.333 to 2.703	0.584
30 min after resuscitation	20	0.460	2.463	0.551	-4.466 to 5.386	0.954
60 min after resuscitation	20	1.865	2.844	0.636	-3.823 to 7.553	1.101

LOA, limits of agreement (MD ± 1.96SD); MD, mean difference; SD, standard deviation of the difference; SEL, standard error of limit; SEM, standard error of the mean difference; SpO_2trachea_, mixed venous oxygen saturation measured through the left main bronchus; SvO_2blood_, oxygen saturation from pulmonary artery samples.
